# Impact of a high-risk multimorbidity integrated care implemented at the public health system in Chile

**DOI:** 10.1371/journal.pone.0261953

**Published:** 2022-01-14

**Authors:** Paula Zamorano, Paulina Muñoz, Manuel Espinoza, Alvaro Tellez, Teresita Varela, Francisco Suarez, Maria Jose Fernandez

**Affiliations:** 1 Centro de Innovación en Salud ANCORA UC, Facultad de Medicina, Pontificia Universidad Católica de Chile, Santiago, Chile; 2 Health Technology Assessment Unit, Center of Clinical Research, Pontificia Universidad Católica de Chile, Santiago, Chile; 3 Department of Public health, Pontificia Universidad Católica de Chile, Santiago, Chile; 4 Analysis and Management of Health Information Unit, Servicio de Salud Metropolitano Sur Oriente, Metropolitana, Chile; 5 Development and management of Patient-Centered care strategies Unit, Servicio de Salud Metropolitano Sur Oriente, Metropolitana, Chile; University of Copenhagen: Kobenhavns Universitet, DENMARK

## Abstract

During recent years, multimorbidity has taken relevance because of the impact of causes in the system, people, and their families, which has been a priority in the health care plan. Interventions strategies and their implementation are still an emerging topic. In this context, Centro de Innovación en Salud ANCORA UC, together with Servicio de Salud Metropolitano Sur Oriente, implemented as a pilot study High-Risk Multimorbidity Integrated Care strategy. This study aimed to evaluate the impact of this strategy in terms of health services utilization and mortality. A cohort study was conducted with high-risk patients with multimorbidity, stratified by ACG^®^, intervened between April 2017 and December 2019. The studied population was 3,933 patients who belonged to similar size and location primary care centers. The impact analysis was performed used generalized linear models. Results showed that intervened patients had a significantly lower incidence in mortality (OR 0.56; 95% CI 0.40–0.77), hospital admissions, length of stay, and the number of hospital emergency consultancies. With the proper barriers and facilitators of a real context intervention, the implementation process allowed the systematization and consolidation of the intervention provided in this study. The training for new roles and the constant implementation support from the Centro de Innovación en Salud ANCORA UC team were essential in the progress and success of the intervention. A complete description of the high-risk intervention strategy is provided to contribute to this emerging topic and facilitate its scale-up. We can conclude that this complex intervention was feasible to be implemented in a real context. The Ministry of Health has taken the systematization and consolidation of the conditions for the national scale-up.

## Introduction

Health systems have been challenged for the last years by the epidemic of noncommunicable diseases (NCDs), delivering in death of 41 million people each year, equivalent to 71% of all deaths globally [1]. This explosion caused by globalization, unhealthy lifestyles, and an aging population, among other things, is affecting mainly middle and high-income countries [2, 3]. Though, putting NCDs approach as a priority in the health plan. Moreover, the COVID-19 pandemic has changed care priorities, abruptly disrupting chronic care, probably deepening this problem [4]. To face this challenge in an effective way, care delivery needs to make the transition from the standard disease approach to patient-centered care with a strong emphasis on person multimorbidity and risk stratification [5, 6].

Multimorbidity is defined as the presence of two or more NCDs in a person, and it is associated with a worse quality of life, higher mortality, polypharmacy, and higher costs in health. Therefore, integrated care comprising a comprehensive person center approach, support in clinical decision-making, self-management support, integrated health information systems, and community participation seems to be today the best approach [7]. Hence, the risk stratification of the Kaiser Permanente Model that segments the chronic population for a more effective care delivery organization. This approach could potentially prevent complications of underlying diseases, reducing the use of health services and personal health costs, keeping sick people under control [8, 9].

Case management services has shown to been essential in the approach of high-risk people with multimorbidity [10]. The professional and collaborative process of assessing, planning, implementing, monitoring, coordinating and evaluating can be adjusted to individual requirements placing personal care on the center [11]. Furthermore, it guarantees timely treatment and follow-up during transition between health services [12]. The potential of this strategy together with person center care has shown that high-risk persons with multimorbidity could be beneficiated having fewer services utilization due to complications associated with NCDs [10].

In 2017, the Centro de Innovación en Salud ANCORA UC (CISAUC), that belongs to the Faculty of Medicine, Pontificia Universidad Católica de Chile works associated with the National Health Fund (FONASA), the Servicio de Salud Metropolitano Sur Oriente (SSMSO). The CISAUC then started designing, piloting and evaluating a High-risk Multimorbidity Integrated Care strategy. The objective was to reorganize health services delivery by implementing an approach based on integrated care, risk stratification, and case management, among others, that can give efficiency to the health system and change the single diagnosis approach. After three years of implementation and evaluation, the CISAUC has systematized the intervention strategies and generated recommendations for its scalability, which today is taking place throughout the country [13].

This study´s objective is to evaluate the impact in high-risk adults with multimorbidity in mortality and health services utilization.

## Materials and methods

We conducted a cohort study with secondary real-world data routinely collected by the Unidad de Analisis y Gestión de la Información (UNAGIS) by the SSMSO between April 2017 and December 2019. We compared seven primary health centers (PHC), where the intervention was implemented with seven control PHC, which maintained the standard model of care based on a diagnostic approach. The UNAGIS, together with the expert team from the CISAUC, selected the control centers according to the covered population (reference of the size of the primary care center) and territorial proximity, being blind to other local characteristics.

The selected population were persons older than 15 years belonging to the SSMSO. This health service is one of the biggest in Chile, and it is in Santiago, in the southeast area. It has a population coverage of more than 1,5 million people and offers primary, secondary, and tertiary care. The eligible population was calculated based on electronic data. They were classified as high-risk with ACG^®^ software by the SSMSO. Data from primary, secondary, and tertiary care, gathering the clinical and demographic history of the patient, were collected to model and predict individual risk over time. A study conducted in Chile showed that using the ACG system for risk classification and the potential use of resource allocation mechanism fitted well in our population [14, 15].

The eligibility process followed three steps. First, they were segmented as high-risk with the following inclusion criteria: ACG RUB (Resource Utilization Band), the two highest categories 4 and 5, two or more chronic conditions, and current consumption of three or more medicines. Second, exclusion criteria were applied from ACG; stage five of chronic kidney failure and transplant immunosuppression. Third, the resulting list of the selected population was sent to each case manager in the PHC, where they performed a final review of the following exclusion criteria: enrollment in palliative care, pregnant women, active cancer diagnosis, severe physical dependence, and drug and alcohol addictions. This process was updated annually for the recruitment of new patients.

The exclusion criteria mentioned above were applied because, in the Chilean health system, these diseases already have enough protocoled offer of health services (16–20). Case management services would add even more services ending in an over-intervention for patients disturbing the expected effects.

After this eligibility criteria process, an average of 37% of high-risk multimorbidity persons were eligible for this intervention. The number of participants was 1,136 intervened and 2,797 control. The matching process was done statistically to analyze the impact outcomes. It followed a 1:2 proportion to have a more significant population. It was done by the UNAGIS given the personal characteristics of each patient, like age, gender, and the number of comorbidities. The loss of patients during the study was 0,3%.

### Clinical intervention

**Fig 1:**
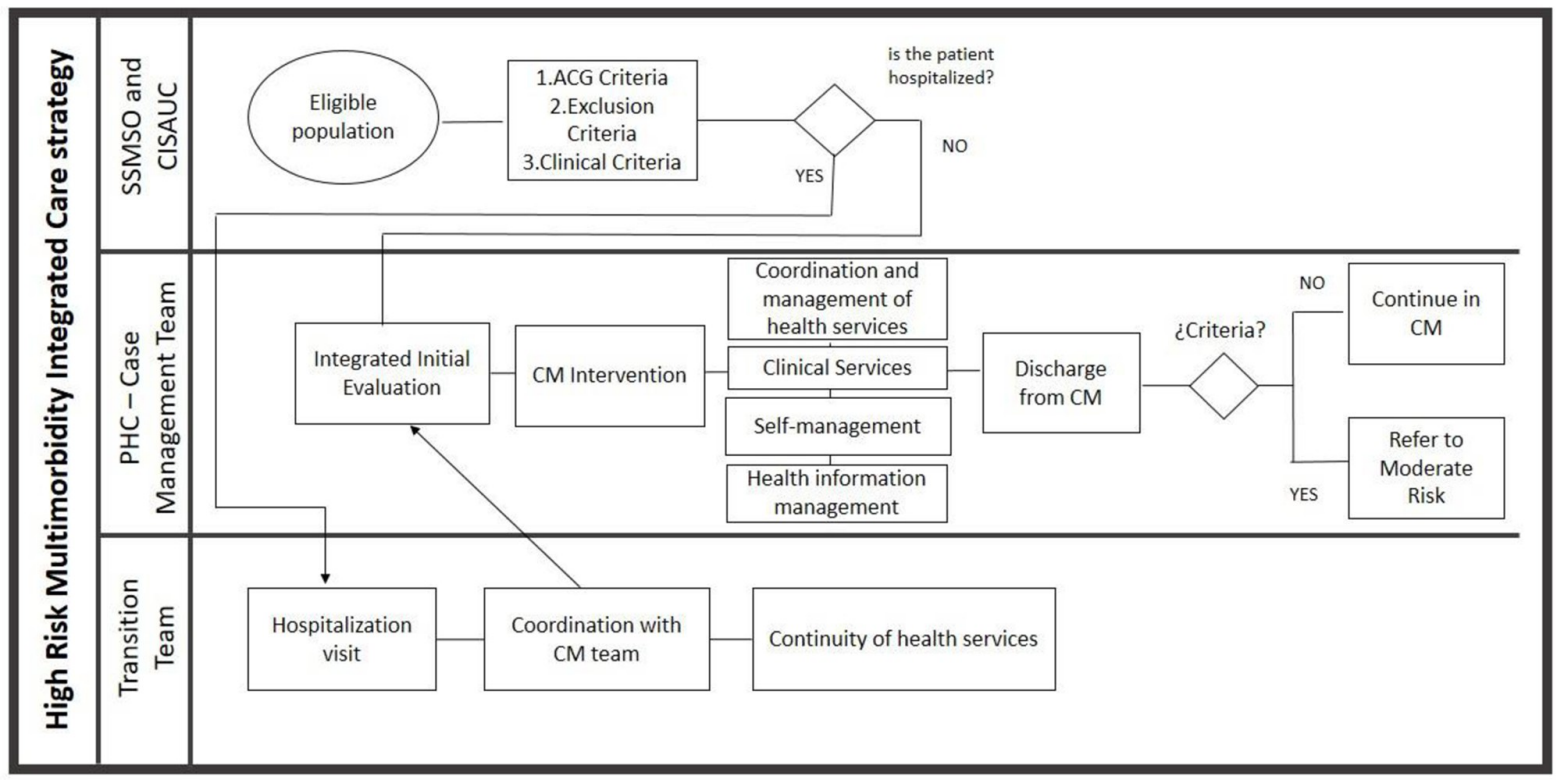
High risk multimorbidity integrated care strategy.

High-risk person data was given to each local team at the PHC. Then they listed them from the highest to lowest ACG weight (assessment that gives relative resource use of each individual) starting a dynamic enrollment from those who had higher ACG weight. The high-risk intervention is shown in Fig 1.

The initial evaluation involved the following activities:

1. *Phone contact*: the objective was to prepare documentation and give essential information for the initial evaluation with the case management couple (nurse case manager and nurse technician) and their assigned General Practitioner. It recollected personal and family data, informed the care case management team assigned, gave case manager contact, and updated laboratory tests when necessary. If this phone contact didn´t reach the patient, the local team assigned a home visit. If the patient didn’t live there or the address did not correspond, the patient was excluded. If the patient was hospitalized after discharge in this first contact, the case manager contacted enrollment.
2. *Application of evaluation instruments*: additional psychosocial assessment instruments were done with the patient. They were: Self-efficacy to manage chronic diseases [16], Morisky and green [17] to evaluate medication adherence and SALUFAM [18] to identify families with more or less risk in developing health complications in their members. This instruments were applied every six months or more, depending on the clinical progress of each patient.
3. *Integrated initial evaluation*: consists of a consultation at the PHC with the case manager nurse and the general practitioner for high-risk patients. They made a bio-psychosocial evaluation, education, and self-management support, incorporating priorities and needs, relevant information on the clinical history of all levels of care to finally define the individualized care plan. For example, establishes agreed to therapeutic goals and clinical follow-up by the case management team with the beliefs and values of the people at the center.

Intervened patients received the following activities and services during the case management period.

1. Coordination and management of health services: clinical care with the multidisciplinary health staff of the PHC was coordinated, assuring that clinical services were provided within the time according to the agreed plan between the health team and the patient. The staff was composed of general physicians, nurses, midwives, nutritionists, psychologists, physiotherapists, and other allied health professionals [19]. Continuity of care was also monitored outside the PHC, like specialist clinical care and other health services done at secondary and tertiary care. Prioritization of these services was arranged when needed to avoid complications from decompensation of NCDs. By last, the transition was monitored from the discharge of hospitalization and emergency consultations to assure continuity of care at the PHC with the case management team.
2. *Clinical services*: performed face-to-face attention for follow-up in nursing care, multimorbidity control and/or home visit. The registration was done in a new protocol implemented in the electronic clinical record system of the PHC that had a particular focus on person center care. This included actualizing patients’ needs and priorities, adjusting to the individualized care plan, clinical evaluation of chronic diseases compensation parameters, self-management support, and activation of family support. Remote services included phone counseling and adherence strengthening of pharmacotherapy. All clinical services included self-management support strategies, face-to-face and remotely.
3. *Self-management additional support*: five group workshops of self-management support were designed together with health teams and the Nursing School of the Pontificia Universidad Católica de Chile. These aim to address the three dimensions of self-management in a multimorbidity approach and the objective of each workshop is described in Table 1. Each workshop was weekly carried out at the PHC with groups of 10–12 persons.
4. *Health information management*: a registration form was designed and implemented for each patient which objective was to consolidate relevant information from different health systems to perform case management. It was used to update the progress on the treatment plan and agreed goals, monitor waiting lists of specialist attention, and register hospitalized patients. For example, it contained a chronological organization of future clinical assessments, phone counseling, and follow-ups activities for each patient, which was crucial for efficient and organized case management.

**Table 1:** Description of self-management workshops offered at the PHC.

Workshops Topics	Objective
My chronic pathologies and their severity	Improve understanding and management of symptoms
My medications, how to get the best out of it	Improve knowledge of the relationship between behaviors and control and clinical parameters achieved
How to take my vital signs and when to consult the health center	Improve decision-making about the use of health services such as emergencies, clinical controls, and nursing support
My chronic pathologies in my house with my family	Promote family support strategies in the process of chronicity
My chronic pathologies: my day-to-day decisions	Increase the perception about the barriers, resources, and strengths for the self-management and implement behavior changes that favor self-management

Regarding discharge from CM, this intervention was defined for a period of time or until their care plan objectives were accomplished. Re-evaluation of the individualized care plan was done every six months with the General practitioner for high-risk patients and the nurse case manager. Transition criteria facilitated the determination of the case management team about the best moment for discharge. If the patient did not meet the established goals, additional transition criteria were defined to protocol the discharge: intervention time without progress in the care plan: 18 months maximum. If the patient met the goals of the treatment established in the initial integrated evaluation, the following criteria were applied: 1) clinical NCD parameters tend to be compensated or compensated within the last six months; 2) absence of hospitalizations or emergency room consultants of decompensation of NCDs in the last three months; 3) improvement in self-efficacy. If they met at least two of the criteria mentioned above, the patient was transferred to more self-managed care, offering moderate interventions conserving their care team.

On the other hand, administrative criteria were defined for patients’ discharge when they were not benefiting from case management. For example, clinical assistance persistently, refusal of case management services, and self-management support or transfer to another primary health center not in this study.

### Implementation process and training

The implementation process had three main steps. First, communication of the sense of urgency and the proposed intervention took place. The CISAUC team, together with the local managers, coordinated several meetings with multidisciplinary teams and decision-makers to explain and involve them in this new challenge. Then training of health teams, especially the new roles and the preparation of the minimum condition for the startup, took place. Second, the implementation started when the first initial evaluation was done in each of the intervened PHC. From then and the next twelve months, frequent visits and advisory were provided from the CISAUC team (Family physician, nurse coordinator and management coordinator) to the local teams, mainly addressing barriers, identifying facilitators, and proposing solutions that allowed the continuity of the implementation to adapt to the reality of each PHC. Finally, and third, monitoring and evaluation activities firmly focused on the systematization of the experience for the future scale-up.

Additional training was provided for the new roles of the case management team. During the first year of implementation, a weekly advisory was provided by an expert team from the Nursing School of the Pontificia Universidad Católica de Chile for case manager nurses, nurse technicians, and general practitioners. It included literature revision, practical cases, and problem-solving of situations they experienced in their daily practice. After that period, these meetings were done once every two months.

From the patient’s perspective, we performed activities such as focus groups and workshops with the patients to get their insights and make the necessary adjustments to improve the clinical intervention and the coordination services. These activities were done in each one of the PHC intervened.

The necessary recourses to implement this pilot were mainly the design of the clinical intervention, for human resources of the case management team to carry out the activities mentioned above, and for monitoring and evaluating. The FONASA provided these resources.

### Outcomes

The impact was evaluated through the following outcomes: (1) number of hospital admissions; (2) length of in-hospital stay; (3) number of consultancies to hospital emergency; and (4) number of consultancies to primary care emergency; (5) death by all causes and (6) drugs use. The following variables were studied and adjusted to the effect estimate: (1) sex; (2) age; (3) number of comorbidities; (4) days exposed; and (5) insurance category. Selection bias was treated with the confounding variables. The insurance category was related to the socioeconomic level of each patient, and it has categorized into eight groups.

Univariate analysis was performed to compare baseline characteristics between the intervention and control groups. Statistical significance was tested using the chi-square test for discrete variables and with a t-test for continuous variables. Impact analysis used generalized linear models to include confounding variables for adjustment. On the binary variable death, we used logistic regression. Moreover, for all discrete counting variables, we explored the best goodness of fit between Poisson and negative binomial models, as well as their “zero-inflated” specifications. In the case of the variable length of stay, we restricted the analysis to those patients who had at least one admission to the hospital. Therefore, the model selected was a negative binomial regression. We chose the zero-inflated negative binomial model for the remaining outcomes because it showed a high proportion of zeros. Statistical analyses were performed in Stata 14.

The study has been approved by the ethical committees of the Pontificia Universidad Católica de Chile and SSMSO, ID190402003: “Centro de Innovacion en Salud ANCORA UC: una contribución al necesario cambio del Sistema de atención en salud”. It did not require informed consent from the participants. It performed only analysis secondary data that will not cause a change in the clinical behavior of the participants, and it will not need to contact the participants for additional information.

## Results

All patients that were intervened in this study received case management services as described above. Baseline characteristic of both groups are presented in Table 2 respectively. The matching process provided by the UNAGIS show the similarity of both groups in terms of baseline characteristics. The following variables had statistically significant differences between groups: sex, age, time of intervention, number of comorbidities and insurance category.

**Table 2:** Baseline characteristics.

	Intervention		Control		p-value
Sex (female)	833 (67,2%)		764 (66,5%)		0,16
Age (SD)	70,2 (11,04)		70,3 (11,75)		0,22
Days exposed (SD)	529,31 (211,80)		542,67 (206,55)		0,06
Number of comorbidities	9,1 (0,1)		8,2 (0,06)		0,06
Insurance category					0,053
• FONASA A	237	21%	494	18%	
• FONASA B	711	62%	1730	62%	
• FONASA C	73	6%	227	8%	
• FONASA D	95	8%	255	9%	
• Dipreca	0	0%	2	0%	
• Capredena	0	0%	9	0%	
• Private	0	0%	3	0%	
• No informed	28	2%	77	3%	

*FONASA: National Health Fund Insurance; Dipreca: Health Insurance of the Police Army; Capredena: National Defense provisional Fund.

In terms of the outcomes, we found that patients enrolled in the High-risk multimorbidity integrated care strategy had a statistically lower chance of dying during the follow-up than those who received the standard management (OR 0.56; CI95% 0.4–0.77). This strategy was also associated with fewer hospital admissions, a shorter stay in the hospital, and a smaller number of consultancies to hospital emergencies (Table 3). All these results were statistically significant, indicating a positive impact of the new model on the health system performance. Furthermore, we also found a significant effect on the utilization of medicaments. Patients under the new model of care were more likely to receive a greater number of drugs than patients in the control arm. This finding is consistent with the fact that intervened patients are under more strict control and follow-up, which usually implies more drugs to control multimorbidity.

**Table 3:** Impact analysis.

Variable	OR /IRR	Estimate (95% CI)	p-value
Death	0.56 (OR)	(0.40–0.77)	0,001
Number of hospital admissions	0.31 (IRR)	(0.20–0.48)	0,000
Length of stay in hospital	0.58 (IRR)	(0.42–0.82)	0,002
Number of consultancies to hospital emergency	0.77 (IRR)	(0.64–0.93)	0,007
Number of consultancies to primary care emergency	0.99 (IRR)	(0.86–1.15)	0,965
Number of drugs	1.15 (IRR)	(1.12–1.19)	0,000

All models adjusted by confounders age, sex, number of comorbidities, baseline risk measured by ACG score, time in the intervention and insurance category. ^a^ Odds Ratios (OR) estimated from Logistic regression; ^b^ Incidence risk ratio (IRR) estimated from zero inflated negative binomial regression; ^c^ Incidence risk ratio estimated from negative binomial regression.

The reorganization and incorporation of new roles and activities were achieved in a process that lasted three years. The implementation process involved a change in how high-risk multimorbidity patients were organized, both in structure and culture. It changed from the organizational structure based on a single diagnosis approach to a multimorbidity one, with all the implications regarding health staff agendas, consultation times, clinical registration, etc. Together, a cultural change started based on the same paradigm, staff used to give health services based on a single diagnostic approach. The challenge of piloting a complex health intervention in the three levels of care required constant support from an external team that could coordinate, monitor, and pushes through the implementation. The CISAUC team had an essential role in giving constant appropriate training and support to preserve the core intervention and work on its sustainability over time.

With the incorporation of new roles, the case management team and the intervention strategies reached their full potential in how health teams reached autonomy to perform the new activities. This process takes six to ten months, where additional external support and permanent peer contact were essential for their success. Although this change had the barrier to entry in a rigid and programmatic system, the principal elements and the core intervention were aligned with the Comprehensive family and community health model, which facilitated the process.

Our study’s caseload for a full-time RN case manager was 93 patients (range 86/118). The variability of the caseload depends mainly on the case manager’s experience in chronic care, management, and network coordination, the complexity of the patients, and the social support provided. On the other hand, the discharge of patients has followed the criteria mentioned in the methodology, and for every patient discharged, a new patient was enrolled to CM. A total of 47% were discharged during the study period (57% of clinical improvement), and 53% continue to receive these services.

About the self-management workshops, they were implemented by all the intervened PHC. The case management team carried them out and other allied health professionals, depending on the topic. To organize and schedule patients, each center organized agendas, space, and materials, for each workshop done monthly. The average of attending patients was nine patients per workshop, and more than 70% of the intervened patients did assist to at least three workshops. The absence or refusal causes were mainly because of transportation problems or hearing losses.

The intervention had acceptance and participation from the patients and their families. As the results show, more than half improved their health condition. It is important to mention that this intervention, in a certain way, avoids waiting lists, speeds up consultations with members of the health team, and avoids having to go to extreme hours of the morning to make an appointment. Added to this, the permanent and defined case management team resulted in patients and their families with greater referred satisfaction and who didn’t want to be discharged from the intervention. Therefore, criteria and definitions were made that allowed better communication and management during patient rotation.

After the experience gained in this training and the need of local health teams, courses were designed and offered by the Nursing School of the Pontificia Universidad Católica de Chile. They contain case management, self-management, motivational interviewing, and adult education, which are now being offered in the scale-up of the Ministry of Health [20].

## Discussion

The purpose of this study was to evaluate the impact of an integrated care model for high-risk patients with multimorbidity in terms of mortality and health services utilization. After a mean follow-up of 529,31 days for the intervened patients and 542,67 days for control patients, we found that intervened patients showed less chance of dying, independent of a set of possible confounders. Furthermore, we also show a statistically significant effect for the intervention in decreasing the incidence of hospital admissions, diminishing the number of consultancies to hospital emergency, and a shorter length of stay in those patients who needed hospital care at least once. In addition, results showed that patients in the intervention group were users of a higher number of medicaments. Though, having implemented case management together with self-management support, remote contact, and an organized follow-up has shown in this study to be essential in reducing mortality and health services utilization.

Along with the health outcomes results mentioned, a detailed description is provided of the intervention to facilitate its future implementation in other jurisdictions. In this, an important matter discussed widely during the piloting process was the caseload of the RN case manager. Although the literature vastly describes the case management process, activities, tools, and others for different clinical settings, the caseload of the RN are still very variable [12, 21, 22]. In this study, we were able to provide training and constant support from the CISAUC, which could be an added value to the integration and development of this new role in the regular staff and activities of the PHC. Furthermore, the conditions that facilitate or hinder its development and performance in the local settings are still a matter to investigate deeper.

Although integrated care is already well described in the literature [23], adding core elements to each group of risk persons with multimorbidity, such as case management for high-risk, seems to be today the best alternative [8, 12]. Furthermore, starting the reorganization of chronic care for high-risk patients can expect positive results in the short term [10]. In contrast, moderate and low-risk groups probably require longer intervention time to show significant results, probably because the reorganization required is deeper and the basis of the intervention is self-management and disease management were results are expected after a longer period [24]. For this reason, we believe that in the scalability of an intervention of this type, it would be desirable to start with interventions that involve groups of high-risk patients.

It is relevant to mention that the remote activities implemented during the piloting were fundamental to facing the pandemic challenge for the COVID-19. Although they were focused only on the high-risk strategy, the implementation of telephone counseling, follow-up, and distance clinical services allowed health teams to become familiar with telemedicine strategies. Cultural and regulatory barriers make their incorporation difficult, as described in the literature [25, 26], and we were able to experience it during the piloting. Barriers range from registration of the activities to the performance of the remote service. This experience was a facilitator in the pandemic’s reorganization of the health system. Moreover, three PHC pilot centers, call centers, and remote services have been provided to continue high-risk multimorbidity integrated care during the pandemic [27].

The implementation and replicability of this intervention in other contexts have not been validated yet, thus the detailed description of the intervention, the implementation process, and the training provided tend to facilitate its economic feasibility and scalability in other territories. A key aspect was that this intervention was aligned with the Chilean PHC principles of the Family and community model. Thus, health teams and decision-makers favored the implementation process and helped mitigate the natural barriers expected from a complex intervention in health. In addition, the set of activities has shown promising results. However, the evaluation of each of the intervention components could provide relevant data.

Future studies should complement the results of this study with measures in health quality of life and a qualitative perspective that can add information from patient in complex interventions. Another challenge it must be addressed is how to measure compensation in patients with multimorbidity. The heterogeneity opens the need for health teams to have a method to measure compensation for multimorbidity in high, medium, and low-risk groups. Finally, an economic evaluation in cost-effectiveness study including a detailed report of health services utilization would be potential information for decision makers facing resources scarcity in the public healthcare system.

## Conclusions

This successful experience in Chile has shown that complex implementations in the public health system are possible and generate good results, which has pushed the national health system into a process of scaling up at the national level [13, 20]. Undoubtedly, this experience serves as the basis to advance health interventions for implementing and evaluating moderate and low risk. In this way, to advance and contribute to the transition that countries have found necessary to address multimorbidity efficiently and effectively. Finally, today the covid-19 pandemic has reorganized health teams and services, which have served as a learning experience for the incorporation and adjustments of future innovations, especially in multimorbidity and comprehensive care person-centered.
